# Resting Energy Expenditure Relationship with Macronutrients and Gestational Weight Gain: A Pilot Study

**DOI:** 10.3390/nu12020450

**Published:** 2020-02-11

**Authors:** Kiley B. Vander Wyst, Matthew P. Buman, Gabriel Q. Shaibi, Megan E. Petrov, Elizabeth Reifsnider, Corrie M. Whisner

**Affiliations:** 1 College of Health Solutions, Arizona State University, Phoenix, AZ 85004, USA; kbvanderwyst@asu.edu (K.B.V.W.); mbuman@asu.edu (M.P.B.); 2 Center for Health Promotion and Disease Prevention, Edson College of Nursing and Health Innovation, Arizona State University, Phoenix, AZ 85004, USA; Gabriel.Shaibi@asu.edu (G.Q.S.); Megan.Petrov@asu.edu (M.E.P.); Elizabeth.Reifsnider@asu.edu (E.R.)

**Keywords:** maternal obesity, maternal nutrition, basal metabolic rate, pregnancy, pregnancy and nutrition, weight gain

## Abstract

Resting energy expenditure (REE) comprises 60% of total energy expenditure and variations may be associated with gestational weight gain (GWG) or maternal diet. The objective of this study was to examine the impact of metabolic tracking on GWG and the association with maternal macronutrients. Pregnant women aged 29.8 ± 4.9 years (78.6% non-Hispanic, White) with gestational age (GA) < 17 week were randomized to Breezing™ (*n* = 16) or control (*n* = 12) groups for 13 weeks. REE by Breezing™ indirect calorimetry, anthropometrics and dietary intake were collected every two weeks. Early (14–21 weeks), late (21–28 weeks), and overall (14–28 weeks) changes in macronutrients and GWG were calculated. The Breezing™ group had a significantly greater rate of GWG [F (1,23) = 6.8, *p* = 0.02] in the latter half of the second trimester. Late (−155.3 ± 309.2 vs. 207.1 ± 416.5 kcal, *p* = 0.01) and overall (−143.8 ± 339.2 vs. 191.8 ± 422.2 kcal, *p* = 0.03) changes in energy consumption were significantly different between Breezing™ and control groups, respectively. Early changes in REE were positively correlated with overall changes in carbohydrates (r = 0.58, *p* = 0.02). Regular metabolism tracking alone did not have an impact on GWG. Early shifts in REE might impact GWG later in pregnancy. Investigation in a larger population from preconception through postpartum is needed.

## 1. Introduction

From 2013 to 2016, overweight and obesity affected 60% and 35% of reproductive-aged women, respectively [1]. While gestational weight gain (GWG) is a normal part of pregnancy, 47% of women gain more than the Institute of Medicine recommended amount of weight [2]. Excessive GWG is associated with a multitude of complications that impact both maternal and neonatal health. These adverse short- and long-term health consequences include preeclampsia, gestational diabetes, postpartum weight retention, fetal macrosomia, neonatal hypoglycemia, and admittance to the neonatal intensive care unit [3,4]. Previous research has demonstrated that greater increases in GWG are associated with decreased physical activity, increased energy intake, higher pre-pregnancy BMI, race/ethnicity, and maternal education [5,6]. Recently, research has focused on the evaluation of resting energy expenditure (REE) across pregnancy to better account for the physiological variability in metabolic adaptations to pregnancy that may also impact GWG [7,8,9,10,11].

REE is the amount of energy expended to maintain basic organ function, respiration, and circulation which accounts for 60%–70% of total daily energy expenditure. Real-time, mobile tracking of REE has become increasingly popular but few devices are able to accurately estimate caloric needs [12]; further, they have rarely been used in pregnant populations [13,14]. Changes in REE during pregnancy have demonstrated an overall increase in caloric needs [8,11,15,16,17] ranging from 13–35%. Recently, the Breezing™ device, a hand-held, Bluetooth-enabled metabolism tracker, that uses indirect calorimetry to measure REE was developed and validated against the laboratory-based Douglas Bag Method [18]. A previously conducted case series of pregnant women that utilized the Breezing™ device demonstrated improved knowledge and awareness of metabolism, weight gain, and caloric intake during pregnancy [19]. This study found unique changes in REE across the participants that were not consistent with predictive equations [19]. It is apparent that REE is a highly variable measure that is impacted by numerous biological (disease status) [20,21] and behavioral (activity level [9], nutrition [10], and sleep [22]) factors. Consideration of REE in conjunction with other biological and behavioral factors may improve dietary and GWG recommendations during a critical period of growth and development.

REE has a strong, positive correlation with total energy, protein, fat, carbohydrates, cholesterol, sugar, and fiber [10]. Total energy intake during pregnancy has been reported to range from approximately 1860 to 2550 kcal/day [23,24,25,26,27]. A recent meta-analysis of 90 studies found that the mean reported intake for total energy, protein, fat, and carbohydrate increased by 184 ± 86 kcal/day, 5.9 g/day, 10.1 g/day, and 17.8 g/day, respectively, from the first to the third trimesters [27]. Although self-reported macronutrient compositions vary among pregnant women [24,25,26,28], an estimated 51% of pregnant women have total energy intakes that exceed daily recommendations, with 19% not meeting carbohydrate and 38% exceeding fat recommendations [29]. It is well known that a high-quality diet during pregnancy is related to better pregnancy outcomes [30,31]; however, there is a paucity of research investigating not only changes in REE during pregnancy but also how REE is influenced by maternal diet and its impact on rate of weight gain.

Obesity during pregnancy has a negative impact on women and their offspring. Because there is a higher proportion of women with obesity prior to pregnancy, greater efforts are needed to minimize the risk of excessive GWG. Assessing and tracking REE during pregnancy might provide a better insight into factors that impact maternal health, including fluctuations in GWG. The purpose of this study was to evaluate how awareness of REE during the second trimester impacted GWG and how variations in REE were associated with maternal macronutrient consumption.

## 2. Materials and Methods

### 2.1. Study Participants and Procedures

**Figure 1:**
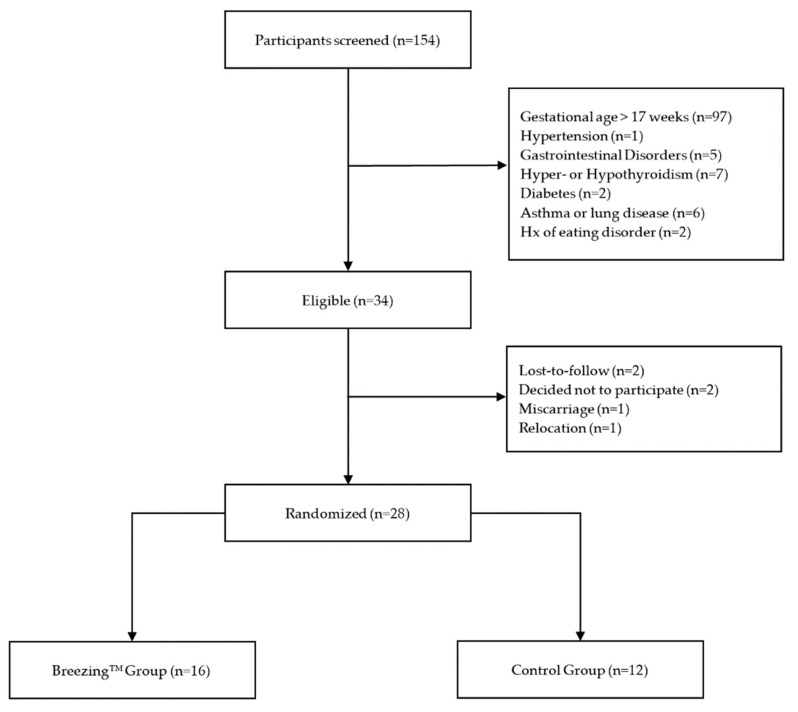
Study Consort Diagram.

This pilot study was a randomized controlled trial of pregnant women (*n* = 28). Women were recruited from Obstetrics and Special Supplemental Nutrition Program for Women, Infants, and Children (WIC) at Adelante Healthcare clinics, social media advertising, and word of mouth referral throughout the greater Phoenix metro area (Figure 1) beginning September 2017 through October 2018. Participants were randomized to the Breezing™ (*n* = 16) or the control (*n* = 12) groups and followed for 13 weeks. Randomization occurred in REDCap through a random number generator. Device use did not allow for blinding of participants or research staff. Seven home visits were conducted by study staff for data collection. Informed consent and demographic data were obtained at the first in-home study visit. All data were compiled in a secure REDCap database. During the duration of the study, study staff frequently contacted participants by phone or text message to check in, answer any questions or address concerns regarding study participation, and to remind them of upcoming appointments.

Nulliparous, primiparous, and multiparous pregnant women with a gestational age <17 weeks and aged >18 years old were eligible to participate. High-risk pregnancies that included the following diseases or conditions were excluded: multiple gestations, fetal growth problems, hypertension, gastrointestinal disorders, malabsorptive diseases, hyper or hypo-parathyroid conditions, HIV, diabetes, asthma/lung disease, cardiac diseases and conditions, current smoker (i.e., women who have smoked 100 cigarettes in their lifetime and now smoke every day or some days), and history of eating disorders. All subjects gave their informed consent for inclusion before they participated in the study. The study was conducted in accordance with the Declaration of Helsinki, and the protocol was approved by the ASU Institutional Review Board. Trial Registration: NCT04131023.

### 2.2. Instruments

#### 2.2.1. Demographic and Health Data

Demographic data collected by study staff included occupation, date of birth, age (calculated as current date minus date of birth), marital status, race/ethnicity, and education. Employment categories were determined using the International Standard Classification of Occupations-08. A health history questionnaire was administered by study staff at each study visit that contained questions about health history and behaviors. Health history items included current gestational age, past medical history, and current use of prescribed or over-the-counter medications and supplements. Medication use included both prescribed and over-the-counter medicines. Prenatal vitamin use was based off self-report and did not include multivitamin use. Health behavior items included current exercise level, dieting status, average per day meal consumption, consumption of caffeinated beverages, average number of drinks per week for caffeinated beverages, and alcohol consumption. Exercise level was defined as the following: sedentary (no exercise), mild (climbing stairs, waking a few blocks, golfing), occasional vigorous (less than 4 times per week for 30 min), and regular vigorous (at least 4 times per week for 30 min or more).

#### 2.2.2. Anthropometric Data

Anthropometrics were collected at every study visit for both groups. Research staff collected height using a portable stadiometer (Seca North America West, Chino, CA, USA; Seca 213) at the first study visit and weight using a portable scale (Seca North America West, China, CA, USA; Seca 876) at each study visit. These measurements were used to calculate body mass index (BMI) as kg/m^2^. Total weight gain was determined as the difference between the participant’s weight at Study Visit 7 and Study Visit 1. Study visit 1 occurred during the first trimester where gestational weight gain is minimal. Rate of weight gain was calculated as the total weight gain in kilograms divided by the number of weeks the participant was followed.

#### 2.2.3. Resting Energy Expenditure (REE)

REE (Breezing™ group only) was determined using the Breezing™ device. The Breezing™ device has been validated against the laboratory-based Douglas Bag Method, which demonstrated a strong significant correlation for VO_2_ (R^2^ = 0.998, *p* < 0.001), VCO_2_ (R^2^ = 0.999, *p* < 0.001), and REE (R^2^ = 0.998, *p* < 0.001) between the two methods [18]. REE measurements were obtained every two weeks during the 13 weeks study to capture variation in metabolic rate. Prior to obtainment of this measurement, participants rested in a seated position for 30 min to reduce the impact of recent physical activity on the REE measurement. The participant remained in a seated position when the measurement was being obtained. They were instructed to breathe in and out of the device for two continuous minutes. The Breezing™ device measures oxygen uptake and carbon dioxide production in order to determine REE. Data were loaded onto an accompanying electronic tablet using a corresponding Breezing™ iOS software application and transmitted electronically to the study investigators. If the device indicated irregular breathing, the study participant would perform the measurement for a second time and the average values were used as the final measurement. After the study participant successfully completed the measurement, study staff reviewed the results with them by showing them the data from the Breezing™ device mobile app. The control participants received the same study visits minus use of the Breezing™ device. No dietary or behavioral counseling or medically-relevant support were provided by study staff to either Breezing™ or control group participants.

#### 2.2.4. Dietary Data

Dietary assessments were completed by study staff using triple pass 24 h dietary recalls [32] at each visit to assess habitual dietary intake during the previous 24 h period. Study staff were trained on how to complete a 24 h dietary recall to ensure standardization in describing and quantifying food and beverage intake. Study staff used a food portion handout to assist in the estimation of food quantity. The triple pass 24 h dietary recall consists of three steps which include a quick list of all foods and beverages consumed in the previous 24 h (unstructured), detailed information on each food and beverage consumed including portion size, time of consumption, and cooking methods, and a final review of all reported information to ensure data accuracy. Each of the 24 h dietary recalls were entered into the Nutrition Data System for Research (NDSR) by the same researcher. The NDSR is a dietary analysis program designed to collect and analyze data from 24 h dietary recalls [33].

#### 2.2.5. Statistical Analyses

All statistical procedures were performed unblinded using SPSS (SPSS 25, Chicago, IL, USA). Demographic characteristics and baseline factors were summarized using counts and percentages for categorical variables, and the mean and standard deviation or median and interquartile range were reported for continuous measures. Overall rate of GWG was calculated as the overall GWG divided by the total study duration in weeks. Early and late rate of GWG were calculated as the difference in GWG between study visits 4 and 1, and 7 and 4, respectively, divided by the total study duration during each respective time period. Changes in REE, energy, and macronutrient (fat, carbohydrates, and protein) consumption were calculated in the same manner. The overall, early, and late changes in rate of and total GWG, and macronutrients were compared between Breezing™ and control groups using a one-way ANCOVA with gestational age at study start, BMI at study visit 1, and maternal education level as covariates. BMI at first study visit was used as a proxy for pre-pregnancy BMI since GWG during the first trimester is relatively low. Correlations between the early, late, and overall changes in REE and GWG (total and rate) and macronutrient consumption were estimated using the Pearson Correlation coefficient after controlling for maternal education and initial BMI. All statistical tests were two-sided, with significance evaluated at *p* < 0.05.

## 3. Results

### 3.1. Demographic and Baseline Data

Of the 34 women who were eligible for the study, 28 were randomized to study groups (16 in the Breezing™ and 12 in the control groups; Figure 1). All 28 women completed the entire study. The median study duration for the control and Breezing™ groups was 13.4 (IQR: 12.9, 14.0) and 13.4 (IQR: 13.3, 14.8) weeks, respectively (Kruskal–Wallis H test, χ^2^ = 1.2, *p* = 0.27). The mean gestational age was 14.8 ± 2.3 weeks at study visit 1, 17.9 ± 2.7 weeks at study visit 2, 20.2 ± 2.5 weeks at study visit 3, 22.4 ± 2.4 weeks at study visit 4, 24.3 ± 2.7 weeks at study visit 5, 26.7 ± 2.8 weeks at study visit 6, and 28.9 ± 2.6 weeks at study visit 7. Complete demographic data and baseline data are provided in Table 1.

Body mass index (BMI) categories of the women at the initial study visit were equally distributed, with 35.7% (*n* = 10) classified as normal weight, 35.7% (*n* = 10) as overweight, and 28.6% (*n* = 8) as obese. The proportion of women that were normal weight (50% vs. 25%), overweight (17% vs. 50%), and obese (33% vs. 25%) for the control and Breezing™ groups, respectively, did not differ (χ^2^ = 3.5, *p* = 0.17).

### 3.2. Intervention Effect on GWG

Table 2 summarizes the rate and total GWG for the study participants. Both groups experienced similar overall rates of [F (1,23) = −1.4, *p* = 0.25] and total [F (1,23) = 1.2, *p* = 0.29] GWG. Compared to the control group, the Breezing™ group had a significantly higher rate of GWG during the second half (i.e., ~21–28 weeks gestation) of the study (F (1,23) = 6.8, *p* = 0.02), but not during the first half (i.e., ~14–21 weeks gestation) of the study [F (1,23) = 0.7, *p* = 0.41]. There was no difference between groups in total GWG during the first half of the study [F (1,23) = 0.5, *p* = 0.47]; however, the Breezing™ group had a non-significant greater total GWG than the control group during the second half of the study [F (1,23) = 4.2, *p* = 0.05].

### 3.3. REE

There were non-significant variations in REE throughout the study [F (6,60) = 0.14, *p* = 0.99]. In total, 80% (*n* = 12/15) of women had an increase in REE between the second and third study visit (mean gestational age: 18 to 20 weeks), with increases in REE ranging from 10 to 350 kcal/day. The proportion of women that experienced an increase (range: 53%–63%) or a decrease (range: 38%–50%) in REE was similar for all remaining study visits. Early changes in REE (72 ± 211 kcals) were relatively small but late changes (128 ± 294 kcals) were nearly twice that of early changes. The mean overall change in REE was 200 ± 316 (range: −340 to 950) kcals. There was an 11.5% increase in total REE between the first and last study visits among the Breezing™ group participants.

### 3.4. Association between REE and Rate of GWG

Early changes in REE were not associated with early changes in the rate of GWG (r = −0.26, *p* = 0.33). Similarly, there was no association between late changes in REE and late changes in GWG (r = −0.18, *p* = 0.55). Likewise, there was no relationship between overall changes in REE and overall changes in GWG (r = 0.01, *p* = 0.96). However, there was a non-significant positive correlation between early changes in REE and late changes in the rate of GWG such that greater increases in REE early in the second trimester were related to greater rates of GWG in the latter half of the second trimester (r = 0.53, *p* = 0.05).

### 3.5. Macronutrient Composition

There were no statistically significant differences for any of the baseline daily dietary intake data (Table 3).

The early, late, and overall changes in consumption of the daily macronutrients among the two study groups are provided in Table 4.

Overall (mean diff = −349.1 ± 150.8, 95% CI: −660.3 to −37.9, *p* = 0.03) and late (mean diff = −379.9 ± 143.9, 95% CI: −676.9 to −82.9, *p* = 0.01) changes in energy consumption were significantly different between the Breezing™ and control groups. There were no statistically significant differences for overall, late, and early changes in protein, carbohydrates, and fat.

### 3.6. Association between REE and Macronutrient Composition

Overall changes in REE were not correlated with overall changes in energy (r = 0.24, *p* = 0.38), fat (r = 0.25, *p* = 0.34), protein (r = 0.36, *p* = 0.17), or carbohydrate (r = 0.03, *p* = 0.91) consumption. Evaluation of early changes in REE with overall changes in carbohydrates revealed a significant, positive correlation (r = 0.58, *p* = 0.02).

## 4. Discussion

The current pilot study provided women with their REE throughout the second trimester and evaluated whether exposure to this information had an impact on GWG. This study found significant differences in the rate of GWG between study groups depending on study time. In the first half of the study, there was no difference in rate or total GWG between the Breezing™ and control group participants. However, this changed in the latter half of the study with the Breezing™ group having a significantly greater rate of weight gain than the control group. This may indicate that GWG during the second trimester might have initially been impacted by the metabolic tracking but that awareness of REE did not have a lasting impact given that late changes in total and rate of GWG among the Breezing™ group exceeded that of the control group. This may be, in part, due to the use of the Breezing™ device only being once every two weeks, or the study being only over a 13 weeks time period which might not have allowed for sufficient exposure to this information. Regardless, to our knowledge, this is the first study that has implemented the use of a real-time metabolism tracker among pregnant women to monitor REE and investigate the impact of REE monitoring on GWG.

In our study, there was an overall increase in REE throughout the study of 11.5%. This is similar to other studies that have assessed REE that found increases ranging from 13% to 35% [8,11,15,16,17]. The large range in changes in REE throughout pregnancy is reflective of the population being studied, the body weight status prior to the study, and the duration of data collection (e.g., preconception to postpartum). The smaller increase in the current study could be explained by the shorter evaluation period. Unlike other studies that found a moderate negative correlation between REE in the 3rd trimester and total GWG [11], we only found a positive correlation between early changes in REE and GWG in the latter half of the study. Our lack of a positive association across the entire study period may be due to the small sample size or large variations in REE at each study visit. Approximately 40% of the women had a decrease in REE which ranged from 10 to 620 kcal/d. Similarly, the women who had an increase in REE had substantial variations ranging from 10 to 350 kcal/d. The current body of work demonstrates the need for a more in-depth evaluation of energy needs during pregnancy and the potential relationship with GWG as some women might experience drastic fluctuations in REE, thereby increasing their risk for excessive GWG or other pregnancy-related health conditions. Future studies may benefit from more regular sampling of REE and monitoring of other behavioral factors (e.g., physical activity) across gestation to fully understand these fluctuations and how they relate to GWG.

The current study also assessed maternal diet composition during pregnancy. Baseline intakes of total fat (69 g/day) for the entire study population were comparable to findings from other studies [26,27,28,34,35,36,37]. Despite percent fat (35.7%) being slightly above the Acceptable Macronutrient Distribution Range, the mean total energy consumption (1690 kcal/day) among our cohort was much lower than other studies (1970–2480 kcal/day) [27,28,35,36]. Likewise, energy from carbohydrates (48%) was slightly lower (31–62 g/day less) than previous reports which ranged from 238 to 269 g/day [26,27,28,35,36]. The percent of calories from protein (17%) was slightly higher than other studies which have reported a range from 14% to 16% [26,27]. However, total daily protein (68.3 g) consumption among the current study participants was lower than the national average (78.1 g) for pregnant women [27]. Despite these minor differences, the overall macronutrient composition of the current cohort of pregnant women resembles that of other studies. When assessing early, late, and overall changes in macronutrients, we observed an overall increase in energy and all macronutrients for the control group whereas the Breezing™ group had an overall decrease; however, time-specific changes varied between groups. Previous studies have found inconsistent findings pertaining to changes in macronutrient intake throughout pregnancy [34,37]. This lack of consensus may be due to variability in baseline dietary composition, differences in the specific time period being studied (i.e., first, second, or third trimester), or homogeneity in cohort demographics. A previous meta-analysis of 90 studies (*n* = 126,242) demonstrated an increase of 184 ± 86 kcal, 5.9 g of protein, 10.1 g of fat, and 17.8 g of carbohydrates per day from the first to the third trimester [34]. The current study demonstrates the variation in maternal diet across pregnancy and the need to have better dietary assessment methods in order to capture accurate changes.

There are several strengths of the current study including the randomized controlled design and overall study adherence. Furthermore, the longitudinal collection of data allowed for evaluation of changes, providing a more complete picture of the fluctuations among these factors during pregnancy. The current study is not without limitations. A major limitation of this study was the small sample size which might have impacted our ability to find significant results. Furthermore, our sample consisted of primarily White, highly educated women which might not be generalizable to the broader population. Additionally, despite greater rate of weight gain in the second half of the study, the Breezing™ group reported an overall decrease in energy intake. This may be in part due to awareness of REE or inaccurate reporting of dietary intake due to group assignment and lack of blinding. Furthermore, there are inherent limitations of a 24 h dietary recall despite using a triple pass approach for data collection. The 24 h dietary recall does not account for day-to-day variation, relies on specific memory of the participant, requires a trained administrator, and may lead to measurement error. Collecting multiple, non-consecutive dietary recalls throughout the study provides a more accurate assessment of habitual diet. Using a triple pass approach that includes both unstructured and structured recall of foods and beverages enhances the accuracy of the data. Finally, the time of REE measurement collection may have resulted in inaccuracies. REE should be measured immediately upon waking prior to consumption of any food or before any exercise. However, this was not possible as the study visits were scheduled at the most convenient times for the study participants. In order to allow for this flexibility but to adjust for potential inaccuracies from measuring REE at variable times, all participants were asked to sit at rest for a minimum of thirty minutes prior to obtaining REE measures.

## 5. Conclusions

Although both groups experienced similar overall and rate of GWG, the Breezing™ group had a significantly greater rate of GWG during the second half of the study compared to the control group. Further, the Breezing™ group had an increase in the overall and late changes in total energy expenditure whereas the control group had a decrease. This study also demonstrated substantial fluctuations in REE, with an approximate 11.5% increase, indicating the need to account for individualized physiological needs during pregnancy and evaluation of their impact on GWG. Early changes in REE correlated with late changes in the rate of GWG demonstrating that early shifts in energy requirements might impact GWG later in pregnancy. Next steps include exploring these relationships with a larger population during the entire course of a pregnancy and the feasibility of integration of the Breezing™ device into healthcare providers’ offices to be used during well-woman and prenatal visits to stimulate discussion about nutritional needs and obtain REE information from preconception through postpartum.

## Figures and Tables

**Table 1:** Participant Demographics and Baseline Anthropometrics, Behavioral, and Prenatal Data.

	Total (*n* = 28)	Control (*n* = 12)	Breezing ™ (*n* = 16)	*p*-Value
Maternal age, mean ± SD	29.8 ± 4.9	29.6 ± 5.9	29.9 ± 4.3	0.86
Race/Ethnicity, % (*n*)				0.36
Non-Hispanic Caucasian	78.6 (22)	75.0 (9)	81.3 (13)	----
Hispanic Caucasian	14.3 (4)	25.0 (3)	6.25 (1)	----
Asian	3.6 (1)	0 (0)	6.25 (1)	----
More than one race	3.6 (1)	0 (0)	6.25 (1)	----
Employment classification, % (*n*)				0.71
Service and sales workers	14.3 (4)	16.7 (2)	12.5 (2)	----
Managerial jobs	3.6 (1)	0 (0)	6.3 (1)	----
Professional	39.3 (11)	50.0 (6)	31.3 (5)	----
Clerical support workers	7.1 (2)	8.3 (1)	6.3 (1)	----
Stay at home mom	35.7 (10)	25.0 (3)	43.8 (7)	----
Marital status, % (*n*)				0.62
Single	3.6 (1)	0 (0)	6.3 (1)	----
Married	85.7 (24)	91.7 (11)	81.3 (13)	----
Partnered/significant other	10.7 (3)	8.3 (1)	12.5 (2)	----
Education level, % (*n*)				0.17
Less than 8th grade	3.6 (1)	0 (0)	6.3 (1)	----
High school/GED	10.7 (3)	25.0 (3)	0 (0)	----
Two-year college	17.9 (5)	8.3 (1)	25.0 (4)	----
Four-year college	32.1 (9)	25.0 (3)	37.5 (6)	----
Post-graduate	35.7 (10)	41.7 (5)	31.3 (5)	----
Weight (kgs), mean ± SD	73.7 ± 16.0	70.3 ± 15.8	76.2 ± 16.2	0.35
Height (cms), mean ± SD	164.1.5	163.5 ± 6.6	164.4 ± 6.6	0.72
Body Mass Index (kg/m^2^), mean ± SD	27.4 ± 5.8	26.3 ± 5.8	28.2 ± 5.9	0.40
Gestational age (weeks), mean ± SD	14.8 ± 2.2	15.3 ± 1.5	14.4 ± 2.7	0.29
Exercise level, % (n)				0.32
Sedentary	14.3 (4)	25.0 (3)	6.3 (1)	----
Mild	57.1 (16)	50.0 (6)	63.5 (10)	----
Occasional vigorous	21.4 (6)	25.0 (3)	18.8 (3)	----
Regular vigorous	7.1 (2)	0 (0)	12.5 (2)	----
Medication use, % (n)				0.69
None	7.1 (2)	8.3 (1)	6.3 (1)	----
1 to 3	71.4 (20)	66.7 (8)	75.0 (12)	----
4 to 6	17.9 (5)	16.7 (2)	18.8 (3)	----
More than 6	3.6 (1)	8.3 (1)	0 (0)	----
Prenatal vitamin use, % (n)	82.1 (23)	83.3 (10)	81.3 (13)	0.89

Independent *t*-tests were performed for all continuous variables to determine whether there were statistically significant differences between groups, and means (SD) are reported. Chi-Square tests were evaluated for all baseline categorical variables to determine whether there were statistically significant differences between groups. Abbreviations: SD = standard deviation; kgs = kilograms; cms = centimeters.

**Table 2:** Mean ± SD Group Differences in the Rate of and Total Gestational Weight Gain (GWG) Among Study Participants.

	Control (*n* = 12)	Breezing™ (*n* = 16)	Total (*n* = 28)	Cohen’s d	*p*-Value
Rate of GWG (kg/week)					
Overall Changes	0.5 ± 0.3	0.6 ± 0.2	0.5 ± 0.2	0.4	0.25
Early Changes	0.6 ± 0.3	0.5 ± 0.3	0.5 ± 0.3	0.4	0.41
Late Changes	0.4 ± 0.3	0.7 ± 0.3	0.6 ± 0.3	1.1	0.02
Total GWG (kg)					
Overall Changes	7.2 ± 2.6	8.1 ± 2.9	7.7 ± 2.8	0.4	0.29
Early Changes	4.2 ± 1.7	3.6 ± 2.0	3.9 ± 1.9	0.3	0.47
Late Changes	3.0 ± 1.4	4.5 ± 2.0	3.9 ± 1.9	0.9	0.05

ANCOVA with maternal education, BMI at study visit 1, and gestational age at study start were performed to compare group differences in rate, and total GWG and means (SD) are reported. Cohen’s d was calculated using means and SDs. Cohen’s d = 0.2 is a small effect, =0.5 is a moderate effect, =0.8 is a large effect. The time period for overall changes were from gestational ages of 14–28 weeks, early changes were from gestational ages of 14–21 weeks, and late changes were from gestational ages of 21–28 weeks. Abbreviations: SD = standard deviation; GWG = gestational weight gain; kg = kilogram.

**Table 3:** Study Group Differences in Baseline Maternal Daily Macronutrient, Fiber, Cholesterol, Caffeine, and Sugar Consumption at the Initial Study Visit.

	Total (*n* = 28)	Control (*n* = 12)	Breezing™ (*n* = 16)	*p*-Value
Total Energy (kcal)	1690.2 ± 495.7	1751.1 ± 493.8	1644.6 ± 508.2	0.58
Total Fat (g)	68.8 ± 29.3	71.0 ± 29.0	67.2 ± 30.3	0.75
Saturated Fat (g) ^a^	22.2 (13.1, 33.4)	18.8 (11.3, 56.6)	26.1 (7.8, 40.7)	1.00
Monounsaturated Fat (g)	25.3 ± 13.0	27.3 ± 14.3	23.8 ± 12.2	0.49
Polyunsaturated Fat (g)	13.0 ± 7.0	12.5 ± 5.7	13.4 ± 8.0	0.73
Cholesterol (mg) ^a^	178.9 (94.5, 283.3)	185.2 (89.4, 273.5)	166.1 (103.3, 292.0)	0.85
Total Carbohydrate (g)	207.8 ± 58.2	219.2 ± 55.9	199.2 ± 60.3	0.38
Total Dietary Fiber (g)	19.6 ± 9.6	22.1 ± 11.2	17.7 ± 8.1	0.23
Soluble Fiber (g)	5.9 ± 2.9	6.6 ± 3.5	5.4 ± 2.3	0.29
Insoluble Fiber (g)	13.6 ± 7.5	15.4 ± 8.5	12.3 ± 6.7	0.28
Total Protein (g)	68.3 ± 24.0	68.6 ± 24.0	68.0 ± 24.7	0.95
Caffeine (mg) ^a^	3.1 (0.0, 47.4)	2.9 (0.0, 88.5)	3.5 (0.0, 44.5)	0.69
Total Sugars (g) ^a^	69.6 (51.7, 107.2)	78.7 (66.0, 104.5)	62.3 (42.9, 121.1)	0.33
Added Sugars (g)	38.9 ± 29.4	32.0 ± 20.7	44.0 ± 34.4	0.26

Independent samples t-tests were performed for all normally distributed data and means (SD) are reported. ^a^ The Kruskal–Wallis non-parametric test was performed for all non-normally distributed data and medians (IQR) are reported. Abbreviations: SD = standard deviation; IQR = interquartile range, defined as the difference between the third and first quartile; kcal = kilocalories; mg = milligrams; g = grams. All data are based on an interviewer-conducted 24 h dietary recall at the initial study visit.

**Table 4:** Mean ± SD Group Differences for Overall, Late, and Early Changes in Daily Macronutrient Intakes (N = 28).

Variable	Control (*n* = 12)	Breezing™ (*n* = 16)	Cohen’s d	*p*-Value
Energy (kcal)				
Overall Changes	191.8 ± 422.2	−143.8 ± 339.2	0.18	0.03
Early Changes	−43.0 ± 766.1	32.2 ± 642.6	0.00	0.79
Late Changes	207.1 ± 416.5	−155.3 ± 309.2	0.23	0.01
Protein (g)				
Overall Changes	13.3 ± 31.0	−9.9 ± 24.0	0.15	0.05
Early Changes	−9.9 ± 19.9	7.4 ± 30.8	0.14	0.06
Late Changes	13.4 ± 33.0	−10.0 ± 22.9	0.15	0.05
Carbohydrates (g)				
Overall Changes	19.3 ± 57.8	−14.5 ± 58.7	0.10	0.12
Early Changes	−2.4 ± 73.6	1.8 ± 87.9	0.00	0.86
Late Changes	19.3 ± 58.0	−14.4 ± 55.8	0.11	0.11
Fat (g)				
Overall Changes	7.4 ± 25.5	−5.5 ± 14.9	0.10	0.11
Early Changes	0.5 ± 60.8	−0.4 ± 28.0	0.00	0.87
Late Changes	7.8 ± 25.2	−5.8 ± 14.5	0.11	0.09

ANCOVA was performed with maternal education and gestational age at study start as covariates to test for group differences. The time period for overall changes was from gestational ages of 14–28 weeks, early changes was from gestational ages of 14–21 weeks, and late changes was from gestational ages of 21–28 weeks. Abbreviations: SD = standard deviation; kcal = kilocalories; g = grams.
